# Optical Genome Mapping as a Tool to Unveil New Molecular Findings in Hematological Patients with Complex Chromosomal Rearrangements

**DOI:** 10.3390/genes14122180

**Published:** 2023-12-05

**Authors:** Nicoletta Coccaro, Antonella Zagaria, Luisa Anelli, Francesco Tarantini, Giuseppina Tota, Maria Rosa Conserva, Cosimo Cumbo, Elisa Parciante, Immacolata Redavid, Giuseppe Ingravallo, Crescenzio Francesco Minervini, Angela Minervini, Giorgina Specchia, Pellegrino Musto, Francesco Albano

**Affiliations:** 1 Hematology and Stem Cell Transplantation Unit, Department of Precision and Regenerative Medicine and Ionian Area (DiMePRe-J), University of Bari “Aldo Moro”, 70124 Bari, Italy; nicoletta.coccaro@uniba.it (N.C.); antonellazagaria@hotmail.com (A.Z.); luisa.anelli@uniba.it (L.A.); francescotarantini14@gmail.com (F.T.); giuseppina.tota@uniba.it (G.T.); maria.conserva1@uniba.it (M.R.C.); cosimo.cumbo@gmail.com (C.C.); elisaparciante@libero.it (E.P.); imma.redavid.ir@gmail.com (I.R.); eziominervini@gmail.com (C.F.M.); minervini.angela@gmail.com (A.M.); pellegrino.musto@uniba.it (P.M.); 2 Section of Molecular Pathology, Department of Precision and Regenerative Medicine and Ionian Area (DiMePRe-J), University of Bari “Aldo Moro”, 70124 Bari, Italy; giuseppe.ingravallo@uniba.it; 3 School of Medicine, University of Bari “Aldo Moro”, 70124 Bari, Italy; specchiagiorgina@gmail.com

**Keywords:** optical genome mapping, cytogenetics, hematologic neoplasms, chromothripsis, complex karyotype

## Abstract

Standard cytogenetic techniques (chromosomal banding analysis—CBA, and fluorescence in situ hybridization—FISH) show limits in characterizing complex chromosomal rearrangements and structural variants arising from two or more chromosomal breaks. In this study, we applied optical genome mapping (OGM) to fully characterize two cases of complex chromosomal rearrangements at high resolution. In case 1, an acute myeloid leukemia (AML) patient showing chromothripsis, OGM analysis was fully concordant with classic cytogenetic techniques and helped to better refine chromosomal breakpoints. The OGM results of case 2, a patient with non-Hodgkin lymphoma, were only partially in agreement with previous cytogenetic analyses and helped to better define clonal heterogeneity, overcoming the bias related to clonal selection due to cell culture of cytogenetic techniques. In both cases, OGM analysis led to the identification of molecular markers, helping to define the pathogenesis, classification, and prognosis of the analyzed patients. Despite extensive efforts to study hematologic diseases, standard cytogenetic methods display unsurmountable limits, while OGM is a tool that has the power to overcome these limitations and provide a cytogenetic analysis at higher resolution. As OGM also shows limits in defining regions of a repetitive nature, combining OGM with CBA to obtain a complete cytogenetic characterization would be desirable.

## 1. Introduction

In the hematologic field, genomic tests are indispensable in the clinical workflow, supporting diagnostic and prognostic classification and guiding therapeutic decisions. In the current state of the art, chromosomal banding analysis (CBA) integrated with fluorescent in situ hybridization (FISH) is considered the gold standard cytogenetic technique with which to identify chromosomal aberrations associated with a clinical significance [1,2,3,4,5,6,7]. However, standard methods present well-known inherent and insuperable limitations that hinder a full insight into the genomic alterations of hematologic neoplasms, such as dependence on cell division in vitro and limited resolving power of about 10 Mb for CBA, and the need for prior knowledge of a specific gene or region to be analyzed, together with the availability of commercially available probes for FISH [8,9,10].

In the last few years, the adoption of Optical Genome Mapping (OGM), a new genome-wide technology, has spread for the analysis of different types of hematological malignancies to detect genomic abnormalities such as structural variants (SVs), copy number variants (CNVs) and aneuploidies of whole chromosomes in a single assay [11,12]. Also known as “Next Generation Cytogenetics”, this method exploits DNA labeling rather than sequencing and is considered a link between techniques based on sequencing and whole chromosome analysis [11,12,13]. Several studies have tested this technology to identify genomic aberrations in both constitutional syndromes and tumors [12,14], demonstrating that OGM is particularly effective in defining complex genomic rearrangements [13,15] such as chromothripsis, consisting of hundreds of genomic rearrangements caused by the shattering of a chromosome and the random reassembly of the generated genomic fragments [16,17,18]. Considering these peculiarities, OGM is frequently applied to the study of hematological diseases, where complex karyotypes are frequent and conventional cytogenetic analysis alone is insufficient. Still, it requires further confirmation by ancillary techniques [13,14,19].

In this study, OGM technology was used to solve the genomic complexity of two hematological neoplasms at the disease onset, previously analyzed with standard cytogenetic techniques, for which these standard methods did not allow a complete analysis of the current cytogenetic aberrations. In case 1, a patient with acute myeloid leukemia (AML) showing chromothripsis, the OGM analysis was fully consistent with classical cytogenetic techniques, highlighting CNVs not detected with standard analyses, helping to more precisely reconstruct the derivative chromosomes generated following the chromothripsis event and to define in depth the breakpoint regions involved. OGM analysis applied to case 2, a non-Hodgkin’s lymphoma case, was only partially in agreement with previous cytogenetic analyses, and helped to better define clonal heterogeneity, overcoming the clonal selection bias due to cell culture of cytogenetic techniques. In both cases, OGM analysis led to the identification of molecular markers, better defining the pathogenesis, classification, and prognosis of the analyzed cases.

## 2. Materials and Methods

### 2.1. Patients

#### 2.1.1. Case 1

In August 2012, a 55-year-old man was admitted to our center with fatigue, dyspnea and epistaxis. His complete blood count (CBC) showed white blood cells (WBC) 11.7 × 10^9^/L, hemoglobin (Hb) 66 g/L and platelets (PLT) 19 × 10^9^/L. A peripheral blood smear showed 53% undifferentiated blasts; a bone marrow (BM) aspirate and biopsy confirmed the same infiltrate of immature, myeloid blasts in the context of a hypercellular marrow. The immunophenotype determined via multiparameter flow cytometry was consistent with AML, FAB M4 subtype. Cytogenetic analysis revealed a rearrangement involving chromosomes 5, 7 and 16. Further molecular analysis showed a *FLT3-TKD* (D835) mutation. Accordingly, the patient was diagnosed with AML, M4 subtype [20].

The patient had primary refractory AML, resistant to a standard “7+3” induction regimen and salvage IDA-FLAG. A third-line therapy—cytosine arabinoside, etoposide and novantrone (MEC)—was attempted, but without hematological remission. Four months after the diagnosis, the patient died due to a fungal infection and consequent septic shock.

#### 2.1.2. Case 2

A 75-year-old patient was admitted to our inpatient clinic for unexplained fever and splenomegaly. His CBC showed WBC 17.8 × 10^9^/L, Hb 99 g/L and PLT 56 × 10^9^/L. His past medical history showed melanoma and squamous carcinoma of the penis, both in complete remission. A serum protein electrophoresis showed the presence of a monoclonal gammopathy of IgM type; moreover, the patient had increased total bilirubin and lactate dehydrogenase values. A BM biopsy was performed, demonstrating the presence of infiltration of a diffuse large B-cell non-Hodgkin lymphoma (DLBCL) cells [17]. Cytogenetic analysis showed a complex karyotype. The patient’s performance status worsened over the next five days; he died during the first cycle of R-CHOP induction therapy due to multiorgan failure.

### 2.2. Chromosome Banding Analysis (CBA)

The karyotype was determined at diagnosis of both cases on BM cells according to standard methods. The BM cells were cultured for 24–48 h, and chromosomes were G-banded with trypsin–Giemsa staining (GTG-banded) according to the recommendations of the International System for Human Cytogenetic Nomenclature [21]. At least 20 metaphases were analyzed.

### 2.3. Fluorescence In Situ Hybridization (FISH) Analysis

FISH analyses were performed on BM samples at the disease onset, using whole chromosome painting (WCP) and bacterial artificial chromosomes (BAC) according to the University of California Santa Cruz (UCSC http://genome.ucsc.edu/; February 2009 release, accessed on 30 November 2023) database. Chromosome preparations were hybridized in situ with probes labeled by nick translation [22].

### 2.4. Multicolor FISH (M-FISH) Studies

Twenty-four color M-FISH probes [20] were applied on slides not previously G-banded, according to the manufacturer’s protocol (SpectraVysion; Vysis, Downers Grove, IL, USA). The WCP probes are directly labeled with the five different fluorophores (SpectrumAqua, SpectrumGreen, SpectrumGold, SpectrumRed, and SpectrumFarRed) in combinatorial labeling. Ten microliters of SpectraVysion reagent were applied to the slides, which were coverslipped, sealed with rubber cement, and placed in Hybrite for denaturation and hybridization with a melting temperature of 68 °C for 2 min, and then a hybridization temperature of 37 °C overnight.

### 2.5. Optical Genome Mapping (OGM) Analysis

The OGM analysis was performed using the Saphyr Bionano Genomics platform (San Diego, CA, USA) [11,21,22,23]. In both cases, BM nucleated cells were used at onset, resuspended in RLT—a lysis and storage buffer containing guanidium isothiocyanate and β-mercaptoethanol.

The samples underwent quality control, and the Ultra-long High Molecular Weight (UHMW) DNA was extracted. UHMW genomic molecules (0.15–2.5 Mb) were fluorescently labeled at specific short nucleotide sequence sites using the DLE-1 enzyme that recognizes a single-stranded DNA pattern of 6 base pairs, allowing a marking density of about 15 labels per 100 Kbp in the human genome, with a labeling periodicity of about every 5 Kbp [11,19]. The labeled DNA was then loaded onto a chip containing nanochannels on which electrophoresis of DNA molecules took place. The molecules were linearized through an imaging process, and the fluorescent pattern of the entire genome was then scanned. Image processing was used to create a consensus map of the analyzed genome [13,23,24]. This consensus map was then compared with a reference genome to identify genomic aberrations. The raw data were analyzed using the two available bioinformatics pipelines: rare variant pipeline (RVP) and “de novo assembly”. These pipelines differ mainly in their ability to detect low-frequency allelic variants: the RVP can identify SVs of between at least 5 kbp and tens of Mbp in length and new genomic fusions that show a Variant Allele Frequency (VAF) threshold of about 5%; it shows coverage of 400X and is most recommended for heterogeneous samples that may have multiple cell clones, such as tumor samples or mosaicism conditions. The “de novo assembly” pipeline creates a de novo assembly of the genome for each chromosome, achieving a low sensitivity for rare events (VAF of at least 15–25%) but detecting smaller SVs, of about 500 bp, compared to RVP [19,25]; it has a coverage of 100X and is the most recommended for germline analysis [13,19]. Then, there is a third analysis tool that is always used independently of the others and that detects CNVs from 500 kb up to aneuploidies, with a VAF of at least 10%; it helps to detect CNVs such as partial aneuploidies and terminal deletions, which are instead lost by the SVs call [13,19].

### 2.6. Expression Analysis by Reverse Transcription-PCR (RT-PCR)

Total RNA was extracted for case 1 from the BM sample at onset using the QIAamp RNA Blood Kit (Qiagen, Valencia, CA, USA) and was quantified with the Qubit fluorimeter. Then, 1000 ng of RNA was retrotranscribed into cDNA using SuperScript VILO retrotranscriptase (Invitrogen, Waltham, MA, USA).

The expression experiment was conducted on 2 uL of cDNA with Taq Platinum DNA polymerase using the following primers: ADCY2/SUGCT_F (ATGCGAGACGCCATCATTG) and ADCY2/SUGCT_R (TGCTGGTTATTTCCTGCTCCCC) for the *ADCY2::SUGCT* fusion gene; BASP1-AS1/ADAMTS2_F (CATACTGGAGGCAGACG) and BASP1-AS1/ADAMTS2_R (AGGTTCTTGACGGCTTTACC) for the *BASP1-AS1::ADAMTS2* fusion gene; TCOF1/CACNG3_F (GCAACCCAAAGAGCAAGAAG) and TCOF1/CACNG3_R (CGAAGAACAGCAGCGTGAC) for the *TCOF1::CACNG3* fusion gene.

Thermal-cycling conditions were 94 °C for 10 min (1 cycle), 94 °C for 30 s, 60 °C for 30 s and 72 °C (40 cycles), 72 °C for 3 min (1 cycle) and a 4 °C hold.

PCR products were purified with the QIAQuick kit (Qiagen, Venlo, The Netherlands), quantified, and sequenced via Sanger sequencing using the SeqStudio™ Genetic Analyzer (Applied Biosystems, Waltham, MA, USA).

### 2.7. Immunohistochemistry (IHC) Experiment on Bone Marrow Biopsy: Myc Protein Expression

Immunohistochemistry was performed on a bone marrow biopsy fixed in 10% neutral-buffered formalin and decalcified with EDTA. Tissue was embedded in paraffin, and a section (4 μm) was deparaffinized, rehydrated through graded alcohols and subjected to antigen retrieval for immunohistochemistry. The section was incubated with rabbit monoclonal anti-c-Myc antibody (clone Y69; 1:100; Abcam, Waltham, MA, USA) with post-primary polymer, blocked with 3% hydrogen peroxide, 3,3-diaminobenzidine (DAB, brown chromogen), and hematoxylin as counterstain. These sequential incubations were performed at room temperature; the section was washed with Tris-buffered saline between incubations. All steps are performed on the automated system. Appropriate negative controls, obtained by substituting the primary antibodies with pre-immune serum, and a positive control were included in the procedure. Myc-IHC was interpreted as a qualitative positive (overexpressed) or negative (not overexpressed) result. A positive result is characterized by a strong Myc nuclear staining in greater than 50% of the neoplastic cells. A negative result is characterized by faint staining in a small percentage of cells (less than 50%).

## 3. Results

### 3.1. Case 1

#### 3.1.1. Standard Cytogenetic Analyses

**Figure 1:**
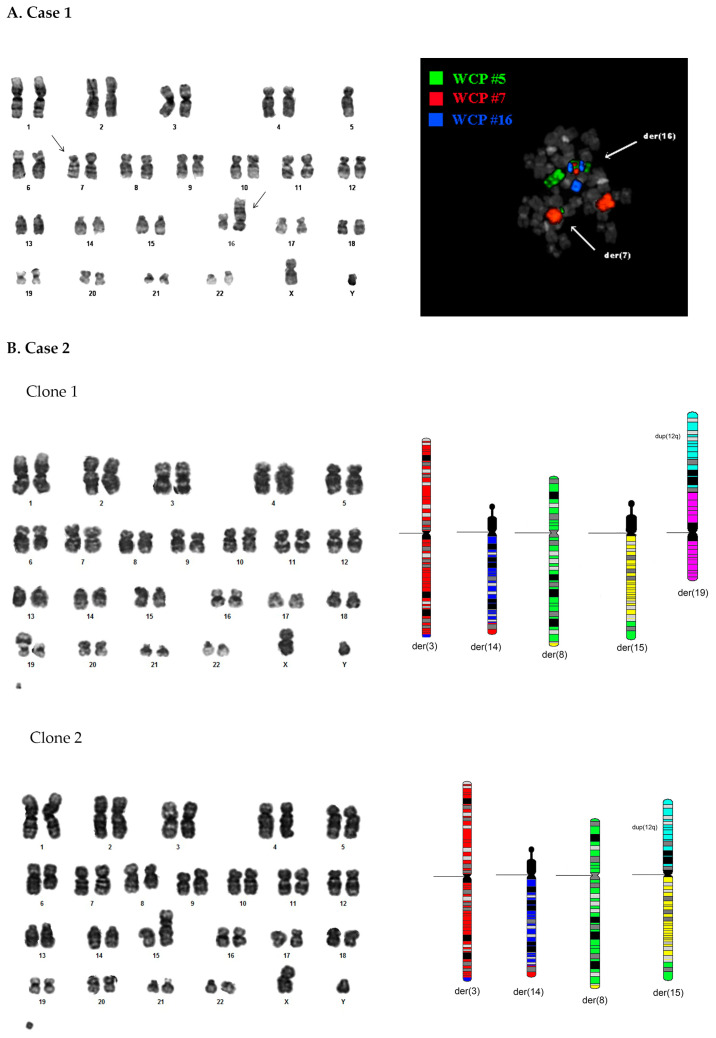
(**A**) Karyotype of case 1 determined via GTG-banding and FISH analysis with WCP probes specific to chromosomes 5, 7 and 16. (**B**) Karyograms by GTG-banding analysis of the two clones detected in case 2, bearing a der(19) and a der(15) chromosome, respectively, and reconstruction of the two cellular clones detected using data derived from FISH analyses.

CBA analysis showed the following karyotype: 45,XY,-5,?t(7;16),der(16)?t(7;16)ins(16;5)[20] revealing a chromothripsis event involving chromosomes 5, 7 and 16 (Figure 1A).

M-FISH analysis confirmed the involvement of chromosomes 5 and 16, but not chromosome 7, in der(16) formation (Supplementary Figure S1A). A further FISH experiment using WCP probes specific for chromosomes 5, 7 and 16 was performed to deepen the information deriving from M-FISH (Figure 1A). It confirmed the monosomy of chromosome 5 and revealed the presence of a der(7) resulting from a t(5;7) translocation in addition to a der(16) resulting from a rearrangement between chromosomes 5, 7 and 16. To precisely define chromosomal breakpoints, repeated FISH experiments were performed with specific BAC clones (Supplementary Figure S1B) selected from the UCSC database belonging to chromosomes 5, 7 and 16 (Supplementary Table S1). They showed a complex rearrangement accompanied by the deletion of large regions of all three involved chromosomes. These data helped to rebuild the complex genomic rearrangement characterized by the “pulverization” of chromosome 5 and the random reassembly of chromosomal fragments in der(7) and der(16) chromosomes (Supplementary Figure S1C). One of the possible mechanisms for generating the complex rearrangement is the one shown: an unbalanced translocation t(5;7) may have occurred first, following which the two chromosomes exchanged pieces of their respective short arms. Subsequently, der(5) chromosome could have been involved in more rearrangements involving chromosome 16; all the involved chromosomes showed the simultaneous loss of large genomic regions. All these events could have co-occurred in a single-cell clone that became predominant, given that this pattern of alterations was observed in all cells.

Based on these genomic data, an attempt was made to investigate the presence of possible molecular alterations without identifying them, probably due to the impossibility of precisely defining the orientation of the pieces of chromosome 5 reassembled on the der(16) chromosome.

#### 3.1.2. OGM Analysis

For each case, reports were generated on the chip metrics (Supplementary Table S3) that contain information on the run and that include, among others, the parameter N50, which represents a value associated with the length of the marked molecules, calculated as a weighted statistical median, such that 50% of the molecules with the indicated length are contained in molecules equal to or greater than this value. This value is a very important parameter that determines the analysis quality. The raw data of the generated molecules (which can be downloaded with files in .bnx format) were then analyzed. For case 1, the analysis was carried out with both the RVP and the de novo assembly pipelines; for case 2, only the RVP was used. The results of the OGM analysis were uploaded to the online server Bionano Access (https://eu.bionanoaccess.com/ProjectBrowser/ProjectBrowserIndex.html, last accessed on 30 November 2023).

**Figure 2:**
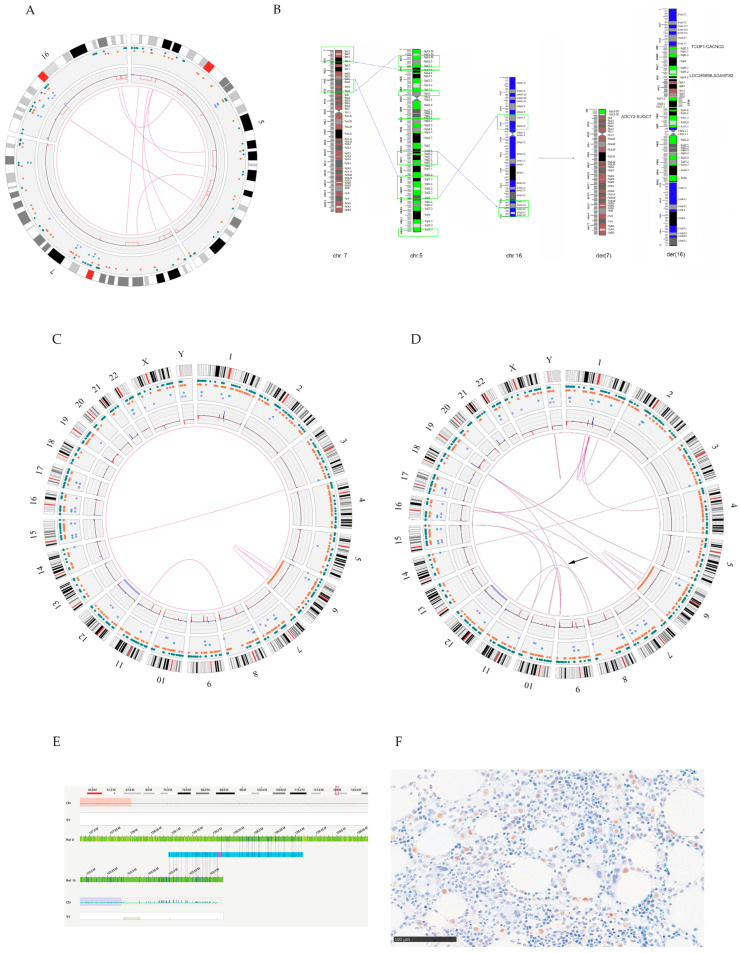
(**A**) Circos plot related to OGM analysis for case 1. The lines indicate the regions involved in the rearrangement. (**B**) Summary of rearrangements detected with OGM and reconstruction of the derivative chromosomes observed in case 1. The green boxes indicate the deleted regions. The blue arrows indicate genomic fusions. The analysis failed to highlight some fusions because they were present in repeated regions. (**C**) Circos plot of case 2 analyzed with stringent filters. (**D**) Circos plot derived from the release of the filter on the masked regions. Most of the fusions found through the analysis affected centromeric and telomeric regions. The arrow indicates the fusion between chromosome 8 and chromosome 15 and the t(8;15) translocation detected in case 2. (**E**) Mapping of one of the fusion molecules generated following the t(8;15) translocation in case 2. (**F**) Immunohistochemical preparation for Myc protein analysis of case 2: note the nuclear staining of the neoplastic cells.

The OGM analysis of case 1 confirmed the data obtained from the FISH experiments and allowed us to highlight additional regions involved by deletions (Figure 2A, Supplementary Table S4). The observation of fusion molecules between regions deriving from different chromosomes also allowed us to redraw the sequence and orientation of the regions involved in generating the der(16) derivative chromosome and, ultimately, to identify more precisely the possible involvement of genes (Figure 2B).

The analysis showed three putative fusion genes: *ADCY2::SUGCT* on the der(7) chromosome and *TCOF1::CACNG3* and *BASP1_AS1::ADAMTS2* on the der(16) chromosome. 

Expression analysis of the three putative fusion genes showed an amplification product only for the *ADCY2::SUCCT* fusion gene. PCR product sequencing confirmed the presence of the fusion transcript originating from the fusion of exon 3 of the *ADCY2* gene (*Adenylate Cyclase 2*) with exon 10 of the *SUGCT* gene (*Succinyl-CoA:Glutarate-CoA Transferase*).

### 3.2. Case 2

#### 3.2.1. Standard Cytogenetic Analyses

CBA analysis showed the following karyotype: 47,XY,del(6q),?del(8q),add(14q),add(15q),add(19p),+m[12]/47,XY,del(6q),?del(8q),add(14q),add(15p),+m[8] (Figure 1B). FISH analyses revealed the presence of a t(3;14) translocation involving *BCL6* and *IGH* loci, respectively. In addition, characterization by repeated FISH experiments with different clones belonging to chromosomes 8, 12, 15 and 19 (Supplementary Table S2) confirmed the deletion of 8q. It showed the presence of two clones, one with a der(19) chromosome resulting from the addition on the short arm of a duplicate segment of chromosome 12q, and the other with a der(15) chromosome resulting from a t(8;15) translocation, to which a duplicate segment of the chromosome 12q was added again on the short arm (Supplementary Figure S1D,E). The two clones characterized by FISH were observed with equal frequencies (about 50%) (Figure 1B).

#### 3.2.2. OGM Analysis

OGM analysis confirmed the presence of the t(3;14) translocation and 12q gain observed by FISH, and of del(6q) observed by CBA, but also showed some discrepancies versus CBA/FISH assessments. The OGM revealed a t(8;11) translocation that escaped previous analyses (Figure 2C). A subsequent FISH experiment confirmed this rearrangement, which consisted of an unbalanced translocation between chromosomes 8 and 11, involving large CNVs (8q deletion and 11q duplication of about 20 Mb) (Supplementary Figure S1F). However, the t(8;11) rearrangement was observed with stringent filters, while CNVs emerged when the filters were released.

It was necessary to eliminate the filter for the masked regions, which masks the repeated regions (Figure 2D, Supplementary Table S5) to confirm the presence of the der(15) and der(19) revealed with the standard techniques. Therefore, t(8;15) could be observed (Figure 2E); on the other hand, OGM analysis failed to fully reconstruct the two derivative chromosomes observed via CBA/FISH; this result was expected, considering the repetitive nature of the regions involved, since der(15) results from the addition on the NOR region of the short arm of an extra fragment of the long arm of chromosome 12, and that der(19) results from the addition of the same duplicate segment of the long arm of chromosome 12 on the telomere region of chromosome 19p. Indeed, the decrease in the stringency of the analysis brought to light new events, such as several losses involving the short arm of chromosome 19 not confirmed by the FISH analysis.

Regarding molecular analysis, the OGM results did not reveal specific new putative fusion genes to be tested, as the emerging rearrangements mainly consisted of large CNVs. In the context of the t(8;15) translocation, *CCAT1* gene involvement was found. This gene is transcribed into a long, non-coding RNA involved in tumor formation and regulates long-range chromosomal interactions, including the locus of the *MYC* oncogene, which maps 500 kb downstream; therefore, it could be speculated that the rearrangement trigger *MYC* dysregulation. To test this hypothesis, as RNA was not available, an immunohistochemical assay was conducted, and the expression of the Myc protein on the BM biopsy was evaluated. This experiment showed an increased Myc protein expression compared to normal (Figure 2F).

## 4. Discussion

The diagnostic process is the crux of care of the hematological patient. It includes screening, detection, diagnosis, and prognosis of the disease and monitoring of the response to treatment. In this context, the need arises to use the most reliable technology, which is advantageous in terms of saving economic resources and time and ease of use. In addition, technological advances have led to the discovery of additional genomic aberrations and complexities that are often undetectable using conventional diagnostic approaches [26,27,28,29,30].

In this study, OGM, a new cytogenetic technology, was used to complete the cytogenetic characterization of two patients with hematological malignancies who showed complex karyotypes for which extensive efforts produced with standard techniques had failed to provide an exhaustive analysis.

In case 1, cytogenetic techniques showed a chromothripsis phenomenon involving chromosomes 5, 7 and 16. The catastrophic event of fragmentation of chromosome 5 produced der(7) derived from the deletion of 7p and addition of 5p, and a der(16) chromosome derived from the addition of genomic regions belonging to both chromosomes 5 and 7. The OGM analysis was fully consistent with classical cytogenetic techniques but highlighted the deletion of chromosome 5 regions not detected by FISH. The information derived from the OGM data allowed us to reconstruct the der(16) chromosome structure more precisely, helping to define the involved breakpoint regions in greater depth and highlighting the presence of three putative fusion genes. However, given that not all fusion genes are transcriptionally active, we conducted additional molecular biology experiments to explore their transcriptional activation state. This further molecular investigation through RNA expression experiments revealed the expression of only one of the three suggested fusion genes: the fusion transcript *ADCY2::SUGCT*.

Both the *ADCY2* and *SUGCT* genes are involved in cellular metabolism and are reported to be altered in cancer. They may contribute to the pathogenesis of the patient’s disease; however, further studies are needed to deepen their actual role in the case study.

The OGM experiment of case 2 produced more small molecules than case 1. However, this circumstance did not appear to affect the quality of the analysis, given that for the same patient, the OGM analysis better clarified the characteristics of the complex rearrangement observed with standard techniques. However, several discrepancies were found between the OGM analysis and CBA/FISH in this case. In detail, the data obtained were different according to the stringency of the used analysis filters. In this regard, it should be noted that Bionano suggests carrying out the analysis with specifically recommended confidence scores and with the masking filters activated; this second filter can be released, if necessary, but not the first, since setting the confidence score to “All” could lead to false-positive calls.

The t(3;14) translocation, the gain of 12q and del(6q), observed with standard techniques and in both clones, has been confirmed. In addition, among the rearrangements called with the recommended filters, a t(8;11) that had not been previously observed was detected and confirmed by a subsequent FISH experiment. This rearrangement is an unbalanced translocation between chromosomes 8 and 11, which involves large CNVs (deletion and duplication of about 20 Mb, respectively). However, the t(8;11) rearrangement is observed with the recommended filters, while CNVs are seen with less stringent “All” filters. This discrepancy confirms and highlights that the CNV tool is less sensitive than SV analysis. In fact, the pipelines that call SVs (de novo and RVP) identify them according to the different labeling patterns (they work based on the labeling of the reference genome), while the pipeline calling CNVs is based on coverage and is independent of labeling. Working differently, pipelines that call SVs are more sensitive than pipelines that detect CNVs, so it would be better to rely on the SV pipeline in cases of discrepancies between the two. In this specific case, the t(8;11) presents a VAF of 0.05 (5%), but the detection limit of the CNV tool is 10%, based on the discrepancy of the data observed by the two analysis tools. However, when carrying out a manual inspection of the region, some red/blue lines are observed in the CNV track, which may indicate the presence of a deletion/duplication.

This same circumstance was also found for other rearrangements observed with FISH that emerged only when performing the analysis with less stringent “All” filters, such as t(8;15). This rearrangement can be observed with recommended confidence scores and “All SVs” in the masking filter, with a VAF of 0.04. The breakpoint on chromosome 15 is very close to the telomere, which could be why the masking filter eliminated this rearrangement.

Other rearrangements found in CBA and FISH experiments were not detected by OGMs, such as the derived chromosomes der(15) and der(19) derived from the addition of duplicate regions of 12q on centromeric and telomeric regions, respectively, of chromosomes 15 and 19 in the two different clones. However, even this evidence was expected, considering the repetitive nature of the involved regions. Since fusion molecules between chromosome 12, 15 and 19 cannot be detected, the OGM analysis does not make translocation calls and fails to indicate where the duplicate region of 12q is located, being unable to detect these rearrangements.

For case 2, it was impossible to refine the characterization of the regions affected by the breakpoints by conducting analysis with the de novo assembly pipeline. The reason is that the de novo assembly pipeline cannot detect events with low allele frequency but only with a VAF of 30–40%, and for case 2 most of the observed rearrangement events have a very low VAF; for example, the VAF of t(8;15) is 0.04.

The results of these analyses have allowed us to confirm that OGM technology undoubtedly provides valid support in the resolution of cases with complex alterations, and is helpful in identifying the molecular actors underlying neoplasms. However, the collected data highlighted the advantages and limits of this technology, some of which are already known and reported in the literature [14,31].

In this study, case 2 showed a clonal selection due to cell culture that led to the definition of prevalent clones effectively present with lower frequencies in the neoplasm. An advantage of OGM, compared to techniques such as CBA and FISH, is that it does not require the preparation of cell cultures and, therefore, is not subject to culture bias. Moreover, unlike other molecular technologies such as next-generation sequencing, there is no amplification phase; this limits the possibility of making an incorrect estimation of variants due to PCR bias, and a more accurate assessment of the frequency of detected aberrations can therefore be obtained. The ability to analyze genomic DNA isolated directly from cells, obviating any bias introduced by amplification or cloning phases, prevents underestimating or overestimating the clones’ prevalence. On the other hand, however, when performing a priori OGM analysis, it is not always possible to detect all sub-clonal anomalies present in a small subset of cells that could instead be detected via FISH or CBA analysis [32]; comparing OGM and FISH data, it has been shown that OGM can detect abnormalities present in at least 10–15% of cells [13,32]. This limitation is critical in both cancer diagnosis, where neoplastic sub-clones may be present, and prenatal diagnosis, where there may be mosaicism. In addition, CBA has the advantage of performing a single-cell analysis and can provide information on clonal architecture. However, the cytogenetic sensitivity for the detection of small cell clones is very low, since usually only 20 metaphases are analyzed, and it detects abnormalities present only in proliferating cells. Therefore, the incidence of aberrations or clone size is further influenced by metaphase selection.

In case 2, OGM could not precisely refine the involved regions, since these occurred at very low allele frequencies. In situations like this, to our knowledge, the only technology that could help to define such regions precisely could be the targeted panel sequencing (TPS). This approach can detect SVs with a VAF of merely 0.5%, with thousands of reads in the targeted region. However, its use is limited to specific needs and does not provide genome-wide information [33].

Another well-described limitation of OGM is related to the localization of breakpoints, as in case 2, where OGM analysis was not able to identify the derived chromosomes der(15) and der(19) because the breakpoints are located within repeated regions or sequences not covered by the OGM analysis, such as centromeric and telomere regions. This is a known limitation: balanced rearrangements involving entire chromosomal arms, such as Robertsonian translocations or end-to-end telomeric fusions, cannot be detected, and isodicentric chromosomes can be misinterpreted [13,19,23,32,34]. For this reason, it would be preferable for OGM testing to always be accompanied by CBA analysis at least.

The evidence of false-negative OGM results, as in case 2, sometimes requires lowering the threshold of the CNVs filter or even removing the filtering to detect some alterations already identified using standard diagnostic techniques. This highlights the fact that an improvement of the CNV algorithm would be desirable in future versions [13]. Conversely, this could expose false positive results, such as identifying false fusions in correspondence with regions that randomly present the same labeling pattern [32].

At the molecular level, the OGM analysis of the two cases studied allowed the detection of potential molecular actors underlying the pathogenesis of the neoplasia in both patients. In particular, in case 2, the demonstration of Myc involvement together with BCL6 represents a crucial finding that could help to potentially better classify the disease as a double-hit lymphoma, also known as high-grade B-cell lymphoma, an uncommon and more aggressive subtype of DLBCL characterized by a worse prognosis [35]. To our knowledge, three other published lymphoma cases have been studied by OGM. However, a detailed characterization has not been reported, as they were included in a large series of heterogeneous hematological samples [36].

A final consideration is that complex karyotypes detected in both reported cases are certainly representative of genomic instability, showing an increased number of break events and chromosomal rearrangements. In the last few years, novel catastrophic mechanisms such as chromothripsis, kataegis, and chromoplexy, causing complex chromosome changes and a condition of genomic chaos, have been described [15]. Moreover, it has been demonstrated that structural and numerical karyotypic alterations can largely influence gene expression; karyotype and transcriptome are in constant flux during cancer cell evolution [37]. These considerations relate to the hypothesis of “karyotype or chromosomal coding”, stating that chromosomal rearrangements influence genomic topology and may regulate gene expression and function [38]. The application of OGM for analyzing these two exemplary cases has demonstrated that this technology is an important aid in providing a map for orientation even when the most complex rearrangements arise.

## 5. Conclusions

In recent years, the rapid development of technological innovations quickly provides enormous quantities of information, building an ever-changing and increasingly detailed view of hematological malignancies. Since the diagnostic framework of many hematological malignancies often requires the analysis of several clinically relevant genomic variants that require a panel of individual assays, use of the innovative OGM analysis system can be an excellent option to provide a rapid, comprehensive analysis of the disease and to overcome some limitations of standard techniques without the need for excessive economic commitment. A critical limit of OGM is its inability to detect SNV, which is mandatory for diagnosing many hematological diseases. Soon, therefore, OGMs could be combined with the sequencing of the entire exome or the entire transcriptome to provide information on all SVs, CNVs, SNVs and/or fusion genes expressed, thus obtaining a more complete picture of all genome alterations occurring in hematological malignancies.

## Data Availability

Data are contained within the article and Supplementary Materials.

## Supplementary Materials

The following supporting information can be downloaded at: https://www.mdpi.com/article/10.3390/genes14122180/s1.
