# Supplementary material for: Optical Genome Mapping as a Tool to Unveil New Molecular Findings in Hematological Patients with Complex Chromosomal Rearrangements
Source: Genes (Basel). 2023 Dec 5;14(12):2180. doi: 10.3390/genes14122180 (PMC10742895; doi:10.3390/genes14122180)
Supplement: Supplementary file 1 [file genes-14-02180-s001.zip › genes-2712908-supplementary/Supplementary data.pdf]

Supplementary Figure S1. A. M-FISH analysis of Case 1 showing a der(16) chromosome generated by the fusion of regions from chromosomes 5 and 16. **B.** FISH experiment with BAC probes showing the cohybridization of probes of chromosomes 5 and 7 on the der(16) chromosome. **C.** Reconstruction of the complex rearrangement revealed in Case 1 using data from the FISH analysis. **D-E.** FISH experiment on Case 2 with chromosome 12 BAC probes shows evidence of the two cellular clones bearing the derived chromosomes der(19) (D) and der(15) (E), respectively. **F.** Mapping of one of the fusion molecules showing the translocation t(8;11) in Case 2.

Supplementary Table S1. Summary of BAC probes used to characterize the complex rearrangement in Case 1.

	BAC clones	Chromosomal band	Genomic position	FISH pattern
**chr5**				
	RP11-978F19	5p15.31	chr5:7,429,830-7,614,518	5, der(7p)
	RP11-956N20	5p15.31	chr5:7,567,934-7,727,960	5, del
	RP11-719E22	5p15.1	chr5:16984072-17,188,450	5, del
	RP11-1031A13	5p15.1	chr5:17,146,132-17,325,568	5, der(16p)
	RP11-782C13	5p13.1	chr5:41,063,958-41,230,605	5, der(16p)
	RP11-1149B6	5p11	chr5:46235286-46384443	5, del
	RP11-1150B10	5q11.1	chr5:49,406,372-49,441,108	5q, der(16q)
	RP11-1115G10	5q21.1	chr5:101398262-101,537004	5, der(16q)
	RP11-614O10	5q21.1	chr5:101,670,005-101,860,287	5, del
	RP11-1141B16	5q22.3	chr5:114,928,040-115,084,720	5, del
	RP11-844I6	5q22.3 - 5q23.1	chr5:115,049,833-115256149	5, der(16p)
	RP11-587C14	5q23.2	chr5:126,191,422-126,379,887	5, der(16p)
	RP11-959B16	5q23.2	chr5:126,699,077-126,893,132	5, del
	RP11-759G10	5q32	chr5:149,562,887-149,746,958	5, del
	RP11-802B10	5q32 - 5q33.1	chr5:149,717,036-149,931,774	5, der(16p)
	RP11-641M21	5q35.3	chr5:178,525,062-178,697,496	5, der(16p)
	5q tel			5, del
**chr7**				
	7p tel			7, del
	RP11-1145K16	7p21.3	chr7:11,526,052-11,672,952	7, del
	RP11-1144D11	7p21.3	chr7:11,670,288-11,817,250	7, der(16p)
	RP11-977N13	7p14.3	chr7:29,586,414-29,769,237	7, der(16p)
	RP11-598L6	7p14.3	chr7:29,718,289-29,895,592	7, del
	RP11-746I21	7p14.1	chr7:41,148,107-41,344,937	7, del
	RP11-727A18	7p14.1	chr7:41,184,324-41,388,108	7, der(7p)
**chr16**				
	16p tel			16, der(16p)

RP11-939O16	16p12.2 - 16p12.1	chr16:24,176,728-24,357,883	16, der(16p)
RP11-933O5	16p12.1	chrchr16:24,368,841-24,578,797	16, del
RP11-909K3	16p11.2	chr16:31,806,425-31,985,101	16, del
RP11-1150K14	16p11.2	chr16:32,364,962-32,506,561	16, der(16p)
RP11-777K14	16q23.2	chr16:80,674,625-80,832,156	16, der(16p)
RP11-1146K12	16q23.3	chr16:80,833,396-80,985,646	16, del
RP11-974F1	16q23.3	chr16:83,935,212-84,116,746	16, der(16q)
RP11-683J18	16q23.3-16q24.1	chr16:84116845-84273389	16, der(16q)

Supplementary Table S2. Summary of BAC clones used for FISH characterization in Case 2. Clones used to carry out FISH experiments to verify rearrangements identified later with the OGM analysis are indicated in bold. *: BAC clone identifying breakpoint position of t(8;11) rearrangement.

BAC clones	Chromosomal band	Genomic position	FISH pattern	FISH pattern
			Clone 1	Clone 2
**chr3**				
RP11-697A9	3q27.3	chr3:187,092,897-187,269,787	3+der(3)	3+der(3)
RP11-632M13	3q27.3	chr3:187,409,272-187,559,392 (BCL6)	3+der(3)+der(14)	3+der(3)+der(14)
**chr14**				
RP11-1145H5	14q32.33	chr14:106,995,083-107,135,809	14+der(14)	14+der(14)
WI2-1337E13	14q32.33	chr14:107,241,651-107,280,235	14+der(3)	14+der(3)
**chr8**				
RP11-838K18 (*)	8q23.1	chr8:106,650,156-106,820,511	8+der(8)	8+der(8)
RP11-624K22	8q23.1	chr8:106,648,457-106,833,333	8+der(8)	8+der(8)
RP11-815K2	8q24.13	chr8:123,010,127-123,277,677	8+der(8)	8+der(8)
RP11-599F24	8q24.13	chr8:123,231,330-123,433,798	8+delete	8+deleto
RP11-737L24	8q24.21	chr8:128,020,102-128,166,538	8+delete	8+deleto
RP11-828N16	8q24.21	chr8:128,171,158-128,363,301	8+der(15)	8+der(15)

RP11-1150B6	8q24.21	chr8:128,385,200-128,551,221	8+der(15)	8+der(15)
**chr11**				
RP11-843H2	11q22.3	chr11:109,706,416-109,928,022	11+der(11)+der(8)	11+der(11)+der(8)
RP11-1077G24	11q25	chr11:134,623,632-134,819,369	11+der(11)+der(8)	11+der(11)+der(8)
**chr15**				
RP11-1122B21	15q26.3	chr15:102,198,592-102,321,240	15+der(15)	15+der(15)
**chr19**				
RP11-878J15	19p13.3	chr19:959,520-1,144,508	19+19	19+der(19)
**chr12**				
RP11-879C9	12q14.2	chr12:63,897,420-64,084,834	12+12	12+12
RP11-1145M7	12q14.2	chr12:64,082,607-64,246,165	12+12+der(15)	12+12
RP11-778M9	12q14.3	chr12:67,347,831-67,518,367	12+12+der(15)	12+12
RP11-947M18	12q14.3	chr12:67,419,690-67,607,491	12+12+der(15)	12+12+der(19)
RP11-1083K9	12q21.2	chr12:79,307,391-79,484,271	12+12+der(15)	12+12+der(19)

Supplementary Table S3. Report on the OGM analysis metrics of the two analyzed patients.

Label	Value Case 1	Value Case 2	Description
Reference	hg19_DLE1_0kb_0labels.cmap	hg19_DLE1_0kb_0labels.cmap	Name of the reference genome this sample was aligned to.
Reference Length	3,095,677,412 bp	3,095,677,412 bp	Total length of reference sequence
Enzyme	DLE-1	DLE-1	Name of the enzyme used in this sample.
Site	CTTAAG	CTTAAG	Recognition sequence of the enzyme used.
N50 (>= 20 kbp)	510.49 kbp	116.63 kbp	N50 of the molecules that are 20kbp or longer)
Total DNA (>= 20kbp)	509.97 Gbp	4,140.12 Gbp	Total amount of DNA from molecules that are20 kbp or longer
N50 (>= 150kbp)	510.49 kbp	244.5 kbp	N50 of DNA molecules that are 150kbp or longer
Total DNA (>= 150kbp)	509.97 Gbp	1,626.28 Gbp	Total amount of DNA from molecules that are150kbp or longer
N50 (>= 150kbp and min sites >=9)	511.99 kbp	246 kbp	Same as other N50 fields, but molecules must have at least 9 labels

Total DNA (>= 150kbp and min sites >= 9)	498.13 Gbp	1,577.77 Gbp	Same as other Total DNA fields, but molecules must have at least 9 labels
Map rate	97 %	75.1 %	Percentage of molecules that are 150kbp or longer mapped to the reference
Effective coverage	156.09	382.76	Total amount of aligned DNA divided by the size of the reference genome times the map rate.
Average label density (>=150kbp)	15.91 /100kbp	14.37 /100kbp	Average number of labels per 100 kbp for the molecules that are 150kbp or longer
Negative label variance (NLV)	6.36	15.25	Percentage of reference labels absent in molecules
Positive label variance (PLV)	2.48	2.51	Percentage of labels absent in reference

Supplementary Table S4. Summary of alterations observed in Case 1.

RVA - SV pipeline calls - translocation_intrachr/interchr

RefcontigID1	RefcontigID2	RefStartPos	RefEndPos	Confidence	Orientation	VAF	Putative GeneFusion
5	7	7570048	40447112	0.76	+/+	0.48	ADCY2-SUGCT
5	5	20956715	115067807	1.0	-/+	0.45	-
5	5	17200614	178558025	1.0	+/+	0.62	LOC285696-ADAMTS2
5	7	20441058	11730728	1.0	+/+	0.44	-
5	7	126368328	29748602	1.0	+/-	0.42	-
5	16	149777716	24328644	0.99	+/+	0.5	TCOF1-CACNG3
5	16	41138792	80766715	1.0	+/-	0.42	-
5	16	101467515	84090545	1.0	+/+	0.48	-

CNV pipeline calls

Chromosome	Start	End	Width (MB)	Type	CopyNumber	VAF
5	19314	2573599	2,55	gain	2	0,08
5	7570048	17202500	9,63	loss	1	0,45
5	20441058	20961572	0,52	loss	1	0,46
5	41138792	46387213	5,25	loss	1	0,43
5	69017825	70304873	1,29	loss	1	0,31
5	101467515	115053027	13,59	loss	1	0,46
5	126368328	149798080	23,42	loss	1	0,43
5	178558025	180550860	1,99	loss	1	0,39

7	10487	11756933	11,73	loss	1	0,46
7	29741974	40442426	10,7	loss	1	0,44
7	61728588	62411082	0,68	loss	1	0,31
7	99928050	102067557	2,14	gain	2	0,08
7	157426955	158398683	0,97	loss	1	0,26
16	1194703	2030546	0,84	gain	2	0,2
16	17946804	18730075	0,78	gain	2	0,11
16	24328644	33813209	9,48	loss	1	0,43
16	80726498	84069287	3,34	loss	1	0,45
16	84594335	89400501	4,38	loss	1	0,55

Supplementary Table S5. List of SVs observed in Case 2 resulting from the release of the masking filter of repeated regions and putative fusion genes generated. SVs already detected with standard techniques are indicated in bold.

RefcontigID1	RefcontigID2	RefStartPos	RefEndPos	Type	VAF	PutativeGeneFusion
1	16	146305863.0	70855669.0	trans_interchr_common	0.61	LOC100288142-HYDIN
1	16	146305863.0	70855669.0	trans_interchr_common	0.61	LOC100288142-HYDIN
1	4	142729351.0	49323558.0	trans_interchr_common	0.55	-
1	2	149015459.0	91915456.0	trans_interchr_common	0.39	-
1	1	120931921.0	149459218.0	trans_intrachr_common	0.32	-
1	1	120931921.0	149798528.0	trans_intrachr_common	0.5	-
1	1	120931921.0	149798528.0	trans_intrachr_common	0.5	-
1	1	143886051.0	206077412.0	trans_intrachr_common	0.32	-
**3**	**14**	**187464477.0**	**106295617.0**	**translocation_interchr**	**0.17**	**-**
5	19	180788315.0	163173.0	trans_interchr_common	0.01	-
6	6	101576997.0	157551891.0	translocation_intrachr	0.35	-
6	6	74568071.0	113035506.0	translocation_intrachr	0.05	-
7	19	10487.0	248092.0	trans_interchr_common	0.05	-
7	16	65523.0	90192279.0	trans_interchr_common	0.01	-
**8**	**15**	**128219996.0**	**102516712.0**	**trans_interchr_segdupe**	**0.04**	**CCAT1-WASH3P**
**8**	**11**	**106772876.0**	**109807959.0**	**translocation_interchr**	**0.05**	**-**
9	16	141148935.0	90186687.0	trans_interchr_common	0.21	-

9	11	141150069.0	177912.0	trans_interchr_common	0.23	-
9	19	141150069.0	244645.0	trans_interchr_common	0.15	-
10	10	46425257.0	51764626.0	trans_intrachr_segdupe	0.17	-
11	15	73015.0	102401582.5	trans_interchr_common	0.13	-
Y	Y	9341795.0	9171732.0	trans_intrachr_common	0.48	TTTY20-TSPY3
Y	Y	9362122.0	9171732.0	trans_intrachr_common	0.48	TTTY20-FAM197Y2;TTTY20-TSPY3
Y	Y	9362122.0	9171732.0	trans_intrachr_common	0.48	TTTY20-FAM197Y2;TTTY20-TSPY3
Y	Y	9371799.0	9298299.0	trans_intrachr_common	0.25	-
Y	Y	9371799.0	9298299.0	trans_intrachr_common	0.25	-
