# Supplementary figures and images for: Optical Genome Mapping as a Tool to Unveil New Molecular Findings in Hematological Patients with Complex Chromosomal Rearrangements
Source: Genes (Basel). 2023 Dec 5;14(12):2180. doi: 10.3390/genes14122180 (PMC10742895; doi:10.3390/genes14122180)

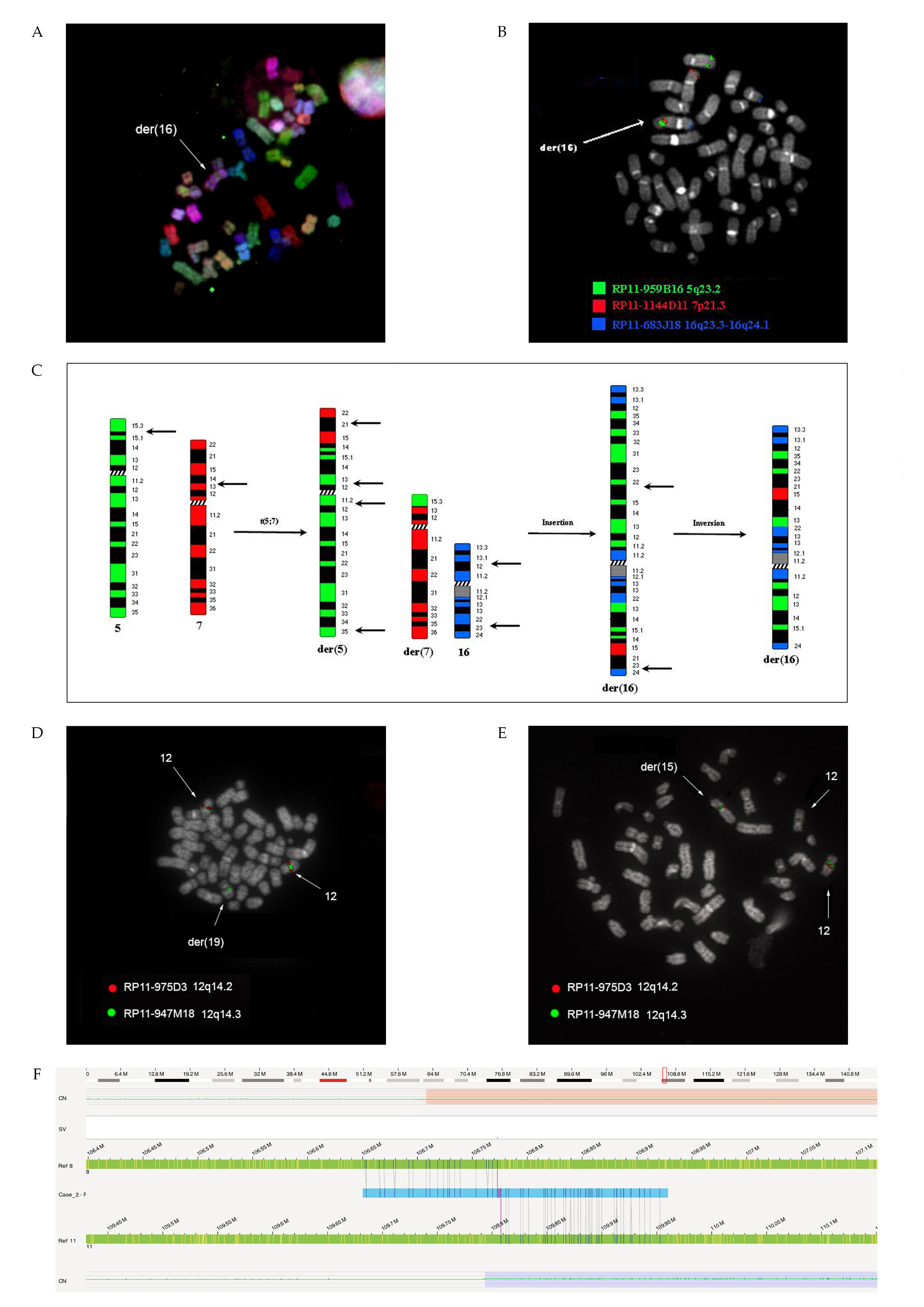

Supplement: Supplementary file 1 [file genes-14-02180-s001.zip › genes-2712908-supplementary/Figure S1.jpg]
